# EEG Feature Extraction Using Evolutionary Algorithms for Brain-Computer Interface Development

**DOI:** 10.1155/2022/7571208

**Published:** 2022-06-29

**Authors:** César Alfredo Rocha-Herrera, Alan Díaz-Manríquez, Jose Hugo Barron-Zambrano, Juan Carlos Elizondo-Leal, Vicente Paul Saldivar-Alonso, Jose Ramon Martínez-Angulo, Marco Aurelio Nuño-Maganda, Said Polanco-Martagon

**Affiliations:** ^1^Facultad de Ingeniería y Ciencias, Universidad Autonoma de Tamaulipas, Ciudad Victoria 87000, Tamaulipas, Mexico; ^2^Intelligent Systems Department, Polytechnic University of Victoria, Ciudad Victoria 87138, Tamaulipas, Mexico

## Abstract

Brain–computer interfaces are systems capable of mapping brain activity to specific commands, which enables to remotely automate different types of processes in hardware devices or software applications. However, the development of brain–computer interfaces has been limited by several factors that affect their performance, such as the characterization of events in brain signals and the excessive processing load generated by the high volume of data. In this paper, we propose a method based on computational intelligence techniques to handle these problems, turning them into a single optimization problem. An artificial neural network is used as a classifier for event detection, along with an evolutionary algorithm to find the optimal subset of electrodes and data points that better represents the target event. The obtained results indicate our approach is a competitive and viable alternative for feature extraction in electroencephalograms, leading to high accuracy values and allowing the reduction of a significant amount of data.

## 1. Introduction

The brain–machine interfaces (BMIs) or brain–computer interfaces (BCIs) are computer systems that allow establishing a direct communication channel between the human brain and a computer [1] to obtain, analyze, and convert brain activity into commands or instructions in real time [2]. The latter is used as a detonator to remotely execute and automate tasks through hardware devices and software applications, the purpose of which is to increase the productivity of people in their work areas or satisfy any need in daily life.

The BCI systems are fed from a constant stream of data corresponding to the readings of the user's brain activity. These data go through a preprocessing stage where a variety of transformations are applied to improve the quality of the data and prepare them for further analysis. This procedure is called feature extraction and consists of identifying patterns in the brain activity that correspond to a certain action (also known as an event) carried out by the user voluntarily or involuntarily. The identified patterns are mapped to specific commands that allow to execute procedures in other digital systems.

As it is well known, the human brain is in constant operation, even when the person is in a state of rest or deep sleep. Consequently, at the time carrying out readings of brain activity, large volumes of data are obtained, being of utmost importance subjecting said data to an exhaustive analysis to find patterns and generate a model that is capable of recognizing such patterns. In this way, the model obtained is integrated into a BCI that operates in real time. In other words, the BCI will be continuously capturing a user's brain activity, which will be evaluated by the pattern recognition model to execute commands.

Nevertheless, the development of this type of BCI has been limited by the factor of time required the analysis and mapping of the information in the EEG [3]. Therefore, the ideal thing is to filter the information contained in the EEG and only keep the necessary data (EEG feature extraction) that allow to typify the operation commands of the BCI.

The problem of EEG feature extraction has been extensively studied and addressed using different techniques. Typically, the process begins with a channel selection phase, which aims to reduce the dimensionality of the data and thus reduce computation times. Then, on the subset of selected channels, the extraction of characteristics is carried out, which are later evaluated with an established metric. A classification of this type of technique is presented in [4] highlighting five main categories, which are filtering, *wrapper*, embedded, hybrid, and manual techniques or with human intervention.

In this work, a method for the feature extraction of EEG based on computational intelligence (CI) techniques is proposed, such as artificial neural networks (ANN) and evolutionary algorithms (EA). The main advantage of the proposed methodology is to use a combination of filtering and *wrapper* techniques for the feature extraction process. The proposed method consists of using an ANN model as a classifier, which is capable of finding features of the EEG signals that allow the identification of one or more types of events. However, the ANN model training process becomes complex due to the variety of information sources (channels) and the volume of data that the EEGs possess. For this reason, an EA is used to optimize the classifier parameters and generate the training and validation sets from the complete EEGs. In this way, the trained and validated ANN model can be used for the detection of events in a real BCI implementation.

The remainder of this document is organized as follows: Section 2 provides an overview of techniques for brain activity monitoring.  Section 3 presents some works of the literature related to feature extraction of EEG.  Section 4 describes the procedure for acquiring EEG signals.  Section 5 presents the steps to perform the training and validation of the classifier.  Section 6 introduces the algorithm to optimize the classification model. The experimental setup and results are shown in Section 7. In Section 8, conclusions and final remarks of the conducted research are given.

## 2. Electroencephalography

Currently, there exists a variety of techniques that allow recording a person's brain activity and are generally used in the field of neuroscience. As it was aforementioned, these techniques can be divided into two categories, invasive and noninvasive. The invasive ones require the implantation of a subcutaneous or intracranial sensor which is responsible for measuring brain activity, while the noninvasive ones make use of sensors (e.g., electrodes or terminals) placed over the scalp to achieve the same purpose. Alternatively, they can be categorized by the type of representation of the bioelectric activity obtained, which can be in the form of an image (2D, heat map, 3D) or time series.

Electroencephalography is a technique used in the medical area that allows for detecting the electrical activity generated by neuronal activity within the brain. The readings of brain activity are done using a set of electrodes, which are positioned in different areas of the scalp and capture the electrical currents produced by neurons [5]. As a result of this, the electroencephalogram (EEG) is obtained, which is a representation of brain activity over some time.

An EEG is made up of *n* time series with *n* being the number of electrodes that were used to measure brain activity. Given their sensitivity with respect to time, EEGs have been used to monitor the effect of anesthesia in surgical procedures, as well as the evaluation and diagnosis of patients with suspected seizures, epilepsy, and some other unusual problems [6].

The main disadvantage of EEGs is that brain activity can be affected by other sources of brain activities produced by the human body itself or external sources in the environment. Nonetheless, its main advantage is that this technique is a noninvasive and low-cost procedure that can be used multiple times in the same patient without any risk.

## 3. Related Works

The problem of EEG feature extraction has been extensively studied and addressed through different techniques. In this section, some of the main work carried out are described, emphasizing the proposed methodology of each one of them. Generally, the process of extraction and selection of characteristics in EEG begins with a channel selection phase, which aims to reduce the data dimensionality and obtain better precision in the classification stage. A classification of this type of techniques is presented in [4] highlighting five main categories, which are filtering, *wrapper*, embedded, hybrid, and manual techniques or with human intervention. Below is some research related to techniques based on filtering and *wrapper*, as they are typically more frequently used.

Filtering techniques operate on subsets of channels obtained by search algorithms, and subsequently, an independent analysis is carried out with techniques based mainly on statistics or probability.

In [7], a statistical model based on the measurement of *Kullback–Leibler divergence* is presented for the selection of the optimal subset of characteristics. In the following paper, the characteristics are extracted by applying an *autoregressive model* (*AR*) and a *common spatial pattern* (CSP-log) algorithm. Afterwards, the selection of the optimal subset and time segments are found with the *Kullback–Leibler divergence* method. A similar approach is proposed in [8] where the *common spatial pattern* (CSP) method and the *discernibility of feature subset* (DFS) metric are used. Wang et al. [9] propose three variants of the algorithm CSP for feature extraction. After this, the features obtained are synchronized with a cross-correlation function.

On the other hand, *wrapper* techniques consist of using a classification algorithm to evaluate the subsets obtained by the search algorithm. In this way, each subset is subjected to a training and testing process in the classifier, where the precision metric dictates the value of the candidate solutions.

Baig et al. [10] present a comparative study of some evolutionary techniques for the selection of characteristics, being the algorithm of differential evolution (DE) as the main object of study. In the first instance, they use a common spatial pattern (CSP) filter for the extraction of characteristics and later generate subsets with the DE algorithm, which are evaluated with classifiers such as linear discriminant analysis (LDA), support vector machine (SVM), *k*-nearest neighbors (k-NN), Naive Bayes classifier (NB), and regression trees. The proposed algorithm is compared with other evolutionary computation algorithms such as particle swarm optimization (PSO), simulated annealing (SA), ant colony optimization (ACO), and artificial bee colony (ABC). As a result of this work, it was found that the performance of the DE algorithm along with SVM was superior with respect to the other analyzed techniques.

In [11], a similar comparison is presented focused on the recognition of emotions in EEG signals, where a scheme consisting of four stages involving preprocessing, extraction, selection, and classification of characteristics is proposed. In the preprocessing stage, signal processing filters are applied for frequency removal and isolation, as well as independent component analysis (ICA) for artifact reduction and data separation into independent components. Feature extraction is performed in three different domains: temporal, frequency, and temporal-frequency by using various methods such as Hjorth parameters, power spectrum density (PSD), and discrete wave transform (DWT), among others. For the feature subsets selection phase, they make use of the ACO, SA, genetic algorithms (GA), PSO, and DE, complemented with a probabilistic neural network (PNN) as a classifier. The results show a higher performance by the DE, PSO, and GA algorithms, and it is concluded that the evolutionary algorithms obtain better results if they work on the combined temporal and frequency spaces.

Lahiri et al. [12] propose a variation of the firefly algorithm (FA) which they call the self-adaptive firefly algorithm (SAFA) to search for the optimal subset of characteristics. Such an algorithm has an extended exploration capacity that consists of self-adapting the step size of each solution close to the position of the best current solution, giving place to a better exploitation of the local neighborhood. The methodology involves a preprocessing of the data for the elimination of artifacts using four IIR filters such as butterworth, Chebyshev type I, Chebyshev type II, and elliptical filter. Regarding the extraction of characteristics, Hjorth parameters and autoregressive adaptive parameters (AAR) were used for the temporal domain, PSD in the case of the frequency domain, and DWT for the time-frequency domain. In addition, a directed acyclic graph support vector machine (DAGSVM) was implemented with three nonlinear kernel functions for task classification, obtaining greater precision with a radial basis function (RBF). The results obtained in this work show that the proposed algorithm has a high degree of effectiveness in the exploration and exploitation of the candidate solutions, avoiding premature convergence problems.

Another evolutionary approach is presented in [13] where the use of a genetic algorithm is proposed for the selection of characteristics related to motor imagination events, specifically the imaginary movement of the hands. For this work, the BCI competition III and IV databases is used, provided by the Berlin BCI group. The data go through a preprocessing phase whose purpose is to improve the quality of the signals, where a discrete Laplacian filter is applied for noise smoothing and a band-pass filter to isolate the bands *μ* and *β* subsequently, the time-frequency domain characteristics are extracted using the short-time Fourier transform short-time Fourier transform (STFT). The GA works with a representation similar to that proposed in [14]; however, the variables encoded in the genes of the chromosome are different. In this case, each chromosome or individual is made up of a number *n* of genes where *n* corresponds to the total number of channels in the EEG, and four variables are encoded in each gene: the channel label, a frequency band, a temporal component index, and a flag that indicates whether the channel is active or inactive.

Moreover, Jin et al. [15] propose a novel feature optimization and outlier detection method for the common spatial patterns algorithm. They use the minimum covariance determinant (MCD) to detect and remove outliers in the dataset then they use the Fisher score to select features. The proposed algorithm was evaluated in terms of iteration times, classification accuracy, and feature distribution using two BCI competition datasets. The experimental results showed that the average classification performance of the proposed method is 12% and 22,9% higher than that of the traditional CSP method.

A great deal of the studies related to the extraction and selection of EEG features are strongly linked to the development of BCI for specific purposes, even though related research is also carried out in the field of medicine. Wen and Zhang [16] propose a methodology for the search of characteristics in EEG with a focus on the detection and analysis of epilepsy. The process consists of performing a feature extraction by applying nonlinear methods, arguing that other methods, such as the fast Fourier transform, are ineffective in this task. Moreover, the combination of multiple algorithms for feature extraction is objected, since this sometimes causes an expansion of dimensionality and redundancy of the same. The authors propose the use of a genetic algorithm for the selection of characteristics in the frequency domain, while the labeling is carried out with multiple classifiers such as *k*-NN, LDA, tree decision, multilayer perceptron (MLP), AdaBoost, and Naive Bayes. The obtained results show a precision range of [0.96, 0.99] in multiclass classification experiments.

## 4. EEG Acquisition

It consists of obtaining the digital signals corresponding to the brain activity of a participant, in other words, the set of EEGs that will later be processed. In the first instance, it is necessary to define the type of event that is intended to characterize, in this work the eye-blink event was used as event. Additionally, it is necessary to define how the participant will receive the stimulus that allows such an event to be triggered. Afterwards, the supervisor must capture and store the brain activity that the participant presents during the execution of the test.

Moreover, the supervisor requires to design a test whose main objective is to generate a stimulus in the participant. In this way, when the person captures the stimulus, it is possible to execute the action that in turn represents the eye-blink event. Generally, a participant must repeat the test on more than one occasion to obtain multiple EEG files, which will later be used to generate the training, validation, and test sets.

In the methodology proposed for the following research paper, it is suggested for the participant to be positioned in front of a computer screen, with the intention that he/she can receive visual stimuli or instructions through multimedia content. In addition to this, another reason why this type of test is recommended is related to taking advantage of the internal or external sensors of the computer, such as the webcam or the microphone. These devices can be useful to obtain other types of information that allow to corroborate the reliability of the carried-out test.

### 4.1. Capture and Storage of EEGs

To capture a person's brain activity, an AURA kit developed by the company Mirai Innovations (https://www.mirai-innovation-lab.com) was used which specializes in the design, development, and research of new technologies was used.

The software included in the AURA kit allows EEG capture and storage to be carried out in CSV format files. However, the AURA software has an option for data retransmission using LSL (Lab Streaming Layer), which must be activated before starting the capture. In such a way the initiative was taken to develop its application to automate some of the processes involved. This includes the capture and storage of data, acquisition of complementary information about the participant, issuance of stimuli or instructions, and video capture with a web camera. This application is linked to the AURA software through LSL and obtains the data in real time from the AURA software. The application consists of a control panel that allows to start, pause and stop the execution of the test with the participant.

## 5. Classification of Events in EEG

It consists of implementing a classification model that is capable of learning and recognizing the distinctive patterns of events in EEGs. Therefore, the model is subjected to a training process where, based on examples, it learns to distinguish such patterns. However, it is necessary to generate training, validation, and test sets starting from the EEG signals.

### 5.1. Data Preprocessing

Up to this point, the information contained in the EEG files is known as raw data or raw EEG, which means that they are files with data without any type of preprocessing or alteration. Despite this, raw EEGs present some level of contamination caused by electrical interference from the biosensing device itself. Hence, it is necessary to preprocess the EEGs obtained so that these are suitable for the feature extraction process.

#### 5.1.1. Signal Filtering

It consists of applying filters for digital signal processing to eliminate noise from the data and modify the amplitude of brain waves by frequency elimination or isolation.

**Figure 1:**
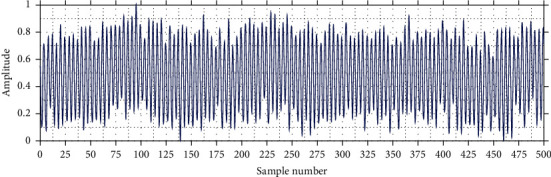
Raw EEG signal.

Figure 1 shows a fragment of raw EEG where it is not possible to appreciate the shape of the brain waves. This is because raw EEGs have contamination generated by electrical interference from the signal amplifier. Such interference generates erroneous signals at the frequency of 50 or 60 Hz, depending on the voltage and frequency of the geographical location.

**Figure 2:**
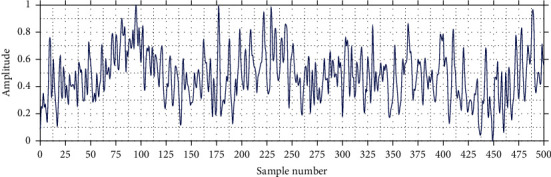
EEG signal with notch filter at 60 Hz.

For this reason, it was decided to apply the notch filter or suppress band, which prevents the passage of signals within the range known as cut-off frequencies. Thus, the electrical interference is suppressed from the signal, making it substantially more readable compared to raw EEG. Furthermore, it allows to visualize the oscillatory patterns of neuronal activity.  Figure 2 shows an EEG fragment after the application of the *notch* filter at 60 Hz.

**Figure 3:**
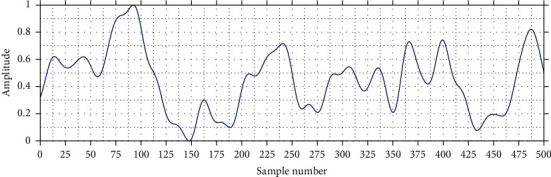
EEG signal with band-pass filter from 1 to 12 Hz.

Moreover, the eye-blink artifact contaminates the low-frequency EEG bands (1–12 Hz) that are associated to hand movements, attention levels, and drowsiness [17]; for this reason, it was decided to use a band-pass filter whose objective was to isolate a certain range of frequencies and eliminate the remaining frequencies. This allows the amplitude and wave period to be modified based on the specified frequency range. In this way, the resulting signal only contains the oscillation patterns corresponding to the frequency range of interest, as shown in Figure 3.

#### 5.1.2. Normalization

The EEG signals have microvolts (*μ*V) as unit of measurement, whose values oscillate in a varied range. Therefore, it was decided to limit the values to the range [0,1] to establish a standard in all the processes involved in the extraction of characteristics. For this, the Min-Max scaling formula which is defined in equation (1) is applied.

(1) $$\begin{array}{c} X^{\prime }=\frac{X-X_{\max}}{X_{\max}-X_{\min}}. \end{array}$$

Where *X* is the set of original values, *X*_min_ and *X*_max_ are the minimum and maximum values, respectively, while *X*′ is the set of scaled values. Then, given a complete EEG of a certain participant, the procedure is applied independently in each of its channels. Therefore, it is necessary to obtain the reference values *X*_min_ and *X*_max_ of a channel before scaling it.

#### 5.1.3. Event Tagging

Event tagging is intended to attach a series of tags or markers to EEG files. Each marker indicates the exact moment in which a certain event occurred during the execution of the test with the participant. In this way, the training and validation sets can be generated from the labeled EEGs, which will be the input of the classification model.

The procedure consists of carrying out an empirical analysis of the files obtained from the test performed, which are the video file and the EEG file. Both files must be analyzed in parallel and synchronously, in such a way that it is possible to determine the instant of time where the participant executes the action corresponding to the event. Therefore, due to the video-EEG time synchrony, it is possible to define the moment in the EEG sample where an event tag should be attached.

**Figure 4:**
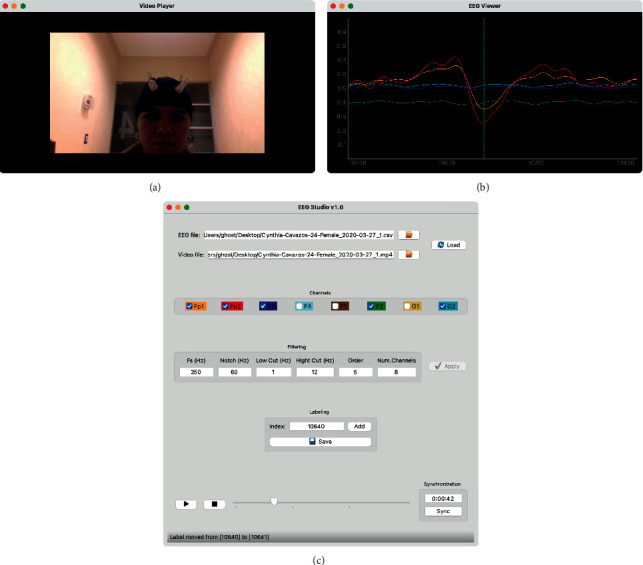
Main window of the developed application for labeling. (a) Video player. (b) EEG viewer. (c) Control panel.

To achieve the previous objective, it was decided to develop an application to speed up the labeling process in the EEG, so that the user only has to load the information of the corresponding files (video/eeg) and the temporal synchronization is carried out automatically. Thus, the remaining procedure is to analyze the video and manually generate the tagging markers. It should be noted that this procedure is of vital importance since if the events are not correctly labeled, the classifier could be taught incorrectly.  Figure 4 shows the application's graphical interface, which consists of a video window (Figure 4(a)), an EEG window (Figure 4(b)), and the control panel (Figure 4(c)).

### 5.2. EEG Segmentation

The operation mode of the BCI systems is in real time since the system works with a constant flow of EEG signals. This implies that the classification model must analyze consecutive segments of the input signals, which have a fixed size and are called windows. Therefore, it is necessary for the EEG obtained from the participants to be segmented in windows.

The purpose of the segmentation, apart from emulating the constant flux of signals, is to generate training, validation, and testing datasets. However, the segmentation process is carried out differently for each set.

**Figure 5:**
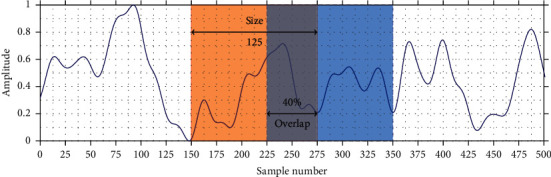
Graphical representation of the segmentation variables. The orange area corresponds to a window at position *i*, while the blue area represents a window at position *i*+1.

The segmentation of EEG is carried out depending on two variables, which are graphically represented in Figure 5 and are described as follows:

- *Size*: It defines the number of consecutive samples (signal points) that make up a window.
- *Overlap*: It indicates the overlap percentage of the *i* window (current) with the *i* − 1 (previous) window.

#### 5.2.1. Training Dataset

**Figure 6:**
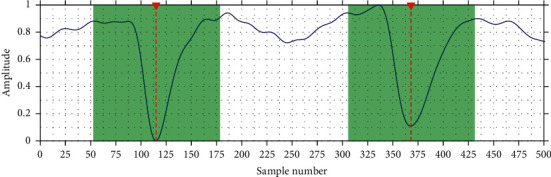
Segmentation of event windows. The green area corresponds to the window and the red vertical lines are the event marker or label.

The segmentation process begins with the location of markers in EEG, which indicates the exact point where an event was recorded in the signal. Each marker serves as the center of a window as shown in Figure 6. Afterwards, the window is extracted and such segment from the original signal is removed, repeating this procedure with each of the markers. Then, the remaining signal (without markers) is segmented in consecutive windows of fixed size and overlapped. Finally, each of the obtained windows (with and without event) make up the dataset for training.

#### 5.2.2. Validation and Test Datasets

For both datasets, a segmentation in translapsed consecutive windows is carried out, by using a control mechanism that allows identifying those windows that have an event marker. Since there is a possibility that the same marker may be present in more than one consecutive window.

### 5.3. Artificial Neural Network Design

It is proposed the use of an ANN-type MLP as a classification model that, once trained, can be used in a real implementation of a BCI.

Nonetheless, some suggestions have been made in the scientific community, and some authors have proposed methods to estimate the number of layers and neurons of the model [18]. Below, some key points that allowed defining the general architecture of the MLP are presented, specifically the distribution of neurons in the hidden layers.

- *Input layer*: The size of the *IL* input layer depends on the window size used in the segmentation process, which must be multiplied by the number of EEG channels.
- *Number of hidden layers*: It is proposed that the MLP has two hidden layers, assuming that the first layer allows to reduce the dimension of the input data, while the second layer is responsible for generalizing or abstracting characteristics of the data.
- *Output layer*: The proposed model was designed to perform a binary classification with each input window. Therefore, the size of the *OL* output layer is 1, since it consists of a single neuron that, based on a threshold function *t*(*x*), it determines the class of an example.

It was considered that the size of the hidden layers of the model should be proportional to the size of the input layer and the output layer. For this, a set of formulas were defined that allow to calculate the number of neurons of each hidden layer. A proportion factor is calculated *κ* as shown in equation (2), where *IL* indicates the size of the input layer and *OL* denotes the size of the output layer. The number of neurons in the first hidden layer *HL*_1_ is defined by equation (3), while the size of the second hidden layer *HL*_2_ is given by equation (4).

(2) $$\begin{array}{c} \kappa =\left\lfloor {\left(\frac{IL}{OL}\right)}^{2}\right\rfloor , \end{array}$$

(3) $$\begin{array}{c} HL_{1}=OL\cdot \kappa^{2}, \end{array}$$

(4) $$\begin{array}{c} HL_{2}=OL\cdot \kappa . \end{array}$$

According to the previously established, the ANN topology has a horizontal pyramid shape where each layer has a smaller size or number of neurons than the previous layer. The neurons of the hidden layers have an activation function known as a rectified linear unit (ReLU) defined in equation (5). On the other hand, the neuron of the output layer has a sigmoid activation function, which is used as a threshold function *t*(*x*), and its definition is presented in equation (6).

(5) $$\begin{array}{c} f\left(x\right)=\max\left(0,x\right), \end{array}$$

(6) $$\begin{array}{c} t\left(x\right)=\frac{1}{1+e^{-x}}. \end{array}$$

### 5.4. Classifier Tuning

The MLP model makes use of the backpropagation algorithm to carry out the learning process during a finite number of times at the training stage. At each time, the average square error MSE (mean squared error) is calculated by

(7) $$\begin{array}{c} \text{MSE}=\frac{1}{n}\sum_{i=1}^{n}{\left(Y_{i}-\hat{Y_{i}}\right)}^{2}. \end{array}$$

Where *Y* is the original label set, $\hat{Y}$ is the set of prediction labels, and *n* is the total number of examples of the training set. In this way, the prediction capacity of the model can be verified, and based on said metric, the backpropagation algorithm adjusts the internal parameters of the model to minimize the error.

### 5.5. Validation of the Classifier

Once the training stage is completed, the model makes predictions on the validation dataset, calculating the *F*_1_ metric which is defined in equation (8). The obtained value represents a harmonic average between the precision metric and the sensitivity metric, which are defined in equations (9) and (10), respectively.

(8) $$\begin{array}{c} F_{1}=2\cdot \frac{PPV\cdot TPR}{PPV+TPR}, \end{array}$$

(9) $$\begin{array}{c} \text{PPV}=\frac{\text{TP}}{\text{TP}+\text{FP}}, \end{array}$$

(10) $$\begin{array}{c} \text{TPR}=\frac{\text{TP}}{\text{TP}+\text{FN}}\text{.} \end{array}$$

Where PPV (positive predictive value) is the precision metric, TPR (true positive rate) is the sensitivity metric, TP (true positive) is the number of true positives, FP (false positive) is the number of false positives, and FN (false negative) is the number of false negatives.

## 6. Optimization of the Classifier

The creation of the training, validation and testing datasets, as well as the number of neurons per layer of the MLP model, are directly dependent on the segmentation variables described in Section 5.2. Up to this point, the assignment of values for these variables is arbitrarily carried out, which implies an extensive test and error process until a combination that shows acceptable results is found.

The current classification model operates with a large volume of data because all the samples of the window of each channel in the EEG are the input of the model. The processing of these data demands a high computational cost, both in terms of memory and in terms of computing time. In turn, this represents a great inconvenience for the operation of a BCI, since the system delay at the time of processing the data and issuing a response must be imperceptible to the user.

However, the characteristic pattern of an event can likely be detected through a subset of samples, and the full window is not necessarily required. It is even possible that the activity registered in more than one channel has no relationship with the event. Therefore, the information of these channels can be completely omitted, allowing the structure of the model to be more compact and efficient. This procedure is known as extraction or selection of features.

Hence, the use of a genetic algorithm (GA) for the optimization of the classification model through the extraction of characteristics is proposed. The procedures involved in the proposed algorithm are described below.

### 6.1. Representation

Each individual is made up of the window overlap variable *x*_1_, which will have a range between 0.1 and 0.9. Additionally, *n* binary variables that allow selecting a *c* active channel subset were used, where *n* is the total EEG channels, for the biosensate sensor was used *n*=8. In addition, other *m* additional binary variables will be included for every channel, which will function as the subset of *f* features of itself, with *m* being equal to the value of the maximum window size variable. Regarding to the latter, it was chosen to set a default value of *m*=250, which is equivalent to windows of one-second long although the algorithm can select a subset of the window.

**Figure 7:**
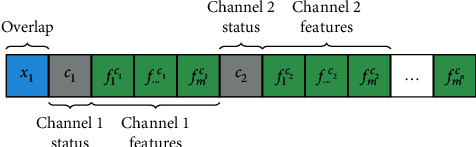
Phenotypic structure of an individual.

If a variable takes the value of 0, the element (channel or feature) that the variable represents will not be considered as input data. Therefore, all those characteristics whose binary variable take the value of 1 will be part of the neuronal network input, as long as the channel variable to which the feature belongs also has the value of 1. In Figure 7, the structure of the individual is shown.

For the representation of the individual, it was decided to use a binary coding based on Gray codes [19, 20], since it has been shown that this kind of coding allows minimum genotypic-level disturbances to generate minimum changes at a phenotypic level.

### 6.2. Objective Function

Given that the GAs require to measure the quality of the individuals of the population (solutions), it is necessary to define a function that allows to quantitatively measure each of the generated solutions by the algorithm. In this case, the best way to determine the quality of a solution regarding the rest, consists of training and validating the classification model. Accordingly, the value of the *F*_1_ metric (described in Section 5.5, equation (8)) obtained in the validation of the model, indicates the quality of the solution. This implies that for each solution generated by the GA, it is necessary to train and validate the proposed classification model.

In the proposed GA, it was decided to use selection by binary tournament, since this type of selection is characterized by being simple and fast, in addition to being one of the most commonly used methods in different evolutionary computing techniques. As a crossing operator for the proposed method, it was chosen to use the uniform cross because its mode of operation involves an exploration of the most aggressive search landscape compared to other types of crosses.

Additionally, it was decided to use uniform mutation, where each allele on the chromosome has the same probability of being inverted. For the calculation of the probability of mutation, it was decided that it was linearly decreasing according to the number of generations, at the beginning of the generations, such probability was ℳ_*i*_=10/*η*, and at the end, it was ℳ_*f*_=1/*η*.

Finally, a *μ*+*λ* selection is used, which consists of selecting only the best *μ* individuals from the current population and the recently integrated new individuals.

## 7. Results

It was established that the event to be characterized was the blinking action, which consists of the closure and opening of both eyelids in a period of time less than or equal to a second. This time interval was established to delimit the duration of the event in question since such action may have a greater duration. Generally, the flashing is considered as an *artifact* of the EEG signals, as well as the breath and the heartbeat [21]. The term *artifact* is used to refer to events (commonly involuntary) of the human body that often obstruct the detection of other events.

A controlled test was designed where a participant must observe a video on the screen of a laptop and has the freedom to blink at any time during video playback, using the capture architecture presented in section 4.1. A total of eight electrodes measure the brain activity of the participant in the areas of the prefrontal cortex Fp1, Fp2, frontal lobe F3, F4, parietal lobe P1, P2, and occipital lobe O1, O2, according to the standard positioning of the 10–20 international system. At the same time, the participant's face is captured through the computer's webcam to later perform the labeling of events in the EEG file.

### 7.1. Description of the Datasets

In the proposed methodology, a total of three datasets are required, which are training, validation, and testing. For this, two capture sessions were carried out with each participant, where the first has an approximate duration of between 8 and 12 minutes, while the second only lasts 4 minutes. Some of the general properties of the acquired EEG are shown in Table 1.

The highest EEG (EEG A) is divided into two parts according to a 70/30 ratio, for subsequently generating the training and validation datasets. That is, the signal segment (starting from sample 1) that encompasses 70% of the events are designated for training, while the remaining signal (with 30% of events) for validation. On the other hand, EEG B is used in its entirety to generate the test dataset.

**Figure 8:**
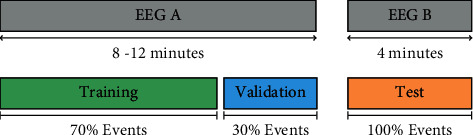
EEG splitting for dataset generation.

In Figure 8, the previously suggested division is shown graphically, while Table 2 shows the distribution of events for the training and validation sets.

### 7.2. Experiment 1: Feature Extraction

This experiment involves carrying out the extraction of characteristics on EEGs obtained from the acquisition phase, to quantify the effectiveness of the proposed method.

From this point, the term Problem will be used to refer to the process of extraction of characteristics in the EEGs of each participant. However, for this experiment, the training and validation datasets will only be used, which are generated from EEG A.

A total of 31 independent executions of the GA described in Section 6 will be performed with the previously presented problems A-1, A-2, A-3, and A-4. The execution parameters of the GA are shown in Table 3.

The configuration of the MLP artificial neural network, such as the activation functions and the size of the different layers, will be carried out according to what is presented in Section 5.3. Although, in this case, the size of the input layer *IL* will not depend on the window size but on the number of active channels and the number of selected features by channel. In terms of the number of training epochs, the model will carry out a total of 10 epochs.

**Figure 9:**
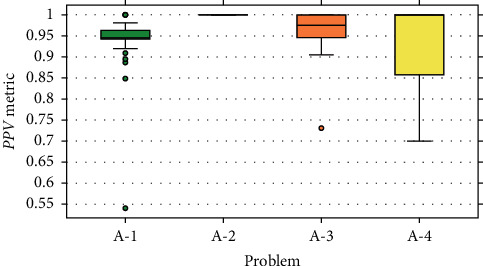
Results for the PPV metric.

**Figure 10:**
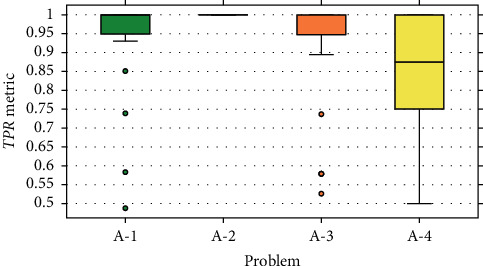
Results for the TPR metric.

**Figure 11:**
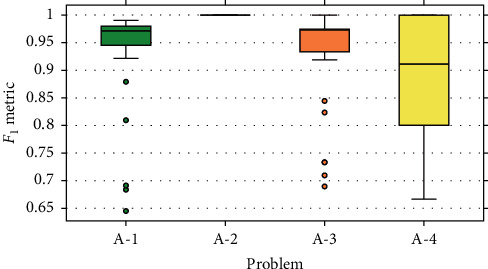
Results for the *F*_1_ metric.

The results obtained from the experiment are presented by boxplot diagrams. In Figures 9–11, the graphs of precision metrics (PPV), sensitivity (TPR), and *F*_1_ are shown, respectively. Each box represents a set of 31 values obtained from the algorithm executions with problems A-1, A-2, A-3, and A-4.

On the other hand, in Table 4, the statistics regarding the feature selection on each problem are presented. Where the *σ* column denotes the value of the standard deviation and the *ϕ* column indicates the percentage of data excluded from the original input. This percentage is estimated according to the average, considering that the full entry consists of 2000 data (window of 250 samples ×8 channels).

For the individual analysis of the results, for each problem, the data of features selection and the data of the evaluation metrics will be presented, both in tabulation format.  Table 5 provides the essential nomenclature for the interpretation of these results.

The data are sorted in ascending order with respect to the *F*_1_ metric and, given that some values are repeated, a second sorting in ascending way was carried out with respect to the total number of selected features. This procedure was performed taking into account the context of the problem in question, where it is seeked to maximize the target function *f*(*x*) ≡ *F*_1_ and minimize the subset of selected features.

On each table, there are three highlighted rows indicating the worst (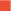), medium (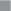), and best (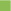) solution out of the 31 executions. Both the worst and the median solution are determined based on the order made, however, to determine which is the best solution, the intervention of an analyst or expert is necessary. Since in some cases, it is possible to know a priori the exact channels that reflect the brain activity of a specific event. Therefore, the best solution should include such sources of information in the subset of selected channels, something that through sorting cannot be secured.

In this particular case, the flashing event has been studied and previously documented. Since the flicker is considered an artifact, it is typically removed from the EEG signals to mitigate interference with other events of interest. Hence, some studies establish that the source of the flashing signals is found in Fp1 and Fp2 channels, by the international electrode positioning system 10–20 [22, 23].

According to the previously presented, three criteria to choose the best solution to each problem were suggested. The first is that the solution must have a value *F*_1_ ≥ 0.90, ensuring a good performance of the classification model. The second criterion is that the solution must include Fp1 and Fp2 channels in the subset of selected channels. Finally, the third lies in that the least number of characteristics selected in total should be counted.

#### 7.2.1. Problem A-1

This problem has a total of 183 events, out of which 128 were used for training and 55 for validation. The boxplot graphs indicate that the values of the evaluated metrics are concentrated above 0.9, and some atypical values are presented in all three cases. According to the statistics in Table 4, the average number of selected features is 605, the amount that represents an average reduction of 69.75% of the input data.

Table 6 shows the number of selected characteristics per channel, in addition to the overlap percentage used in the segmentation of windows. It should be noted that in most executions, the found overlap percentage was greater than 70%.

The worst solution obtained a value of *F*_1_=0.64 with an overlap of 24%, and a total of 477 selected features of the Fp1, Fp2, F4, and P2 channels. Instead, the best solution reached a value *F*_1_=0.96 by selecting 249 features of the Fp1 and Fp2 channels, and an overlap of 82%. Translated to percent, in the best scenario, a reduction of 87.55% of the input data is achieved.

#### 7.2.2. Problem A-2

The boxplot graphs corresponding to the evaluation metrics show null data dispersion because all the values reached 1.0. Therefore, this implies that each of the examples of the validation set was correctly classified. It should be noted that this problem has a total of 218 events, from which 153 were intended for training and 65 for validation.

The statistics in Table 4 indicate an average reduction of 70.66% of the data, based on a mean of 586.68 selected features. In Table 7, the results of the selection of features are presented, where it is observed that an overlapping percentage of over 80% was found in the 31 executions.

Since all the solutions reached a value *F*_1_=1.0, the choice of the best and the worst solution falls (partially) in the total of selected features. The worst solution selects 924 characteristics of seven channels, except for only the P2 channel. While the best solution selects a subset of 358 features of Fp1, Fp2, and P1 channels, achieving a decrease of 82.1% of the input data. In both cases, the solutions operate with an overlap of 87%.

#### 7.2.3. Problem A-3

This problem has a total of 66 events, of which 46 were assigned for training and 20 for validation. The results shown in the boxplot graphs indicate that the values of the metrics are focused mostly above 0.9, with some exceptions considered as atypical values. On the other hand, the statistics presented in Table 4 reveal that the average number of selected features is 437.9, the amount that represents a reduction of 78.1% of the input data.

Table 8 shows the information on the selection of features, where it is possible to notice that 25 of the 31 solutions have an overlap window above 80%.

The worst solution uses an overlap of 30% and brings together a subset of 713 features of six channels (Fp1, Fp2, F3, P1, P2, and O1), reaching a value of *F*_1_=0.68. In contrast, the best solution obtained a value *F*_1_=0.97 with an overlap of 86% and selects a subset of 240 features of the Fp1 and Fp2 channels. Therefore, the input data are reduced by 88%.

#### 7.2.4. Problem A-4

In this problem, there is a total of 27 events, of which 19 were designated for training and eight for validation. The boxplot graphs corresponding to the evaluation metrics show a considerable dispersion in the values obtained, as well as the absence of anomalous data. The highest dispersion comprises the [0.5, 1.0] range and belongs to the sensitivity metric (TPR). The statistics in Table 4 indicate that, on average, the data are reduced by an 87% according to a mean of 251.48 selected features.

The information presented in Table 9 reveals that all solutions completely omit the characteristics of the O1 channel, while the F3, F4, P1, P2, and O2 channels are only selected in few occasions. Moreover, in 14 of the 31 executions, overlap percentages of less than 35% were found, while the rest reached percentages greater than 70%.

Both the worst and the best solution operate with an overlap of 11%. However, the worst solution has a subset of 264 features, selected from Fp2 and P2 channels. With this configuration, the solution obtained a precision *PPV*=1.0 and a sensitivity TPR=0.5, resulting in a value *F*_1_=0.66. On the contrary, the best solution works with a subset of 238 features, selected from Fp1 and Fp2 channels. In this case, a value of *F*_1_=0.93 is achieved with a precision of PPV=1.0 and a sensitivity of TPR=0.875, while the subset of selected features represents a reduction of 88.1% regarding the input data.

#### 7.2.5. Discussion of the Experiment

Based on the boxplot graphs from Figures 9–11, we can conclude that the proposed method presented a better performance at the time of extraction of features with problem A-2. In the 31 executions, a perfect classification was carried out on the validation assembly, and therefore, in the three evaluated metrics, a value of 1.0 was obtained. In the opposite case, the least efficient performance is presented with problem A-4, where it is evident that there is a high variability in the values of the evaluated metrics. On the other hand, from problems A-1 and A-3, the highlighted ones reveal a similar behavior, where in both scenarios the concentration of values occurs in the [0.9, 1.0] range, and they are the only problems where atypical values are presented.

A factor that significantly contributed to obtaining these results was the number of events present in the datasets of each problem. Based on the information presented in Table 1, problem A-2 has the highest number of events compared to the other problems. This allows that, during the training stage, the MLP classification model can efficiently learn the event's pattern. As a result, all predictions on the validation dataset are correct. On the contrary, the deduction related to the results of problem A-4 is that the number of events is insufficient to perform good training. This causes the predictions of the model over the validation dataset to be inconsistent, giving way to obtaining varied results.

However, even though the number of events in problem A-1 is approximately 2.7 times greater than problem A-3, the results obtained from both problems have a certain degree of similarity. This can be appreciated in the boxplot graphs of Section 7.2, especially those that correspond to the TPR (Figure 10) and *F*_1_ (Figure 11) metrics. It is strongly believed that this behavior is due to the stability or consistency of the characteristic pattern of the event. That is to say, the action executed by the participant was replicated multiple times without presenting drastic changes between replicas. In this case, the blinking action can be affected when the person manifests visual fatigue or sleep during the test.

**Figure 12:**
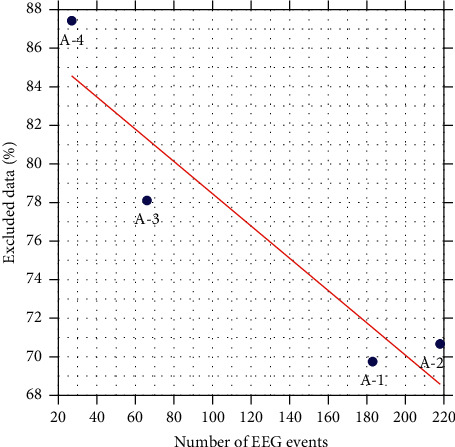
Negative correlation between the number of EEG events and the average percentage of excluded data. The red line represents the trend.

The previous hypothesis is supported on the existing relationship between the number of events in an EEG (Table 1) and the average percentage of excluded data (Table 4). According to the Pearson correlation coefficient, there is a linear dependence *ρ*=−0.93 that indicates a strong negative correlation, as shown in Figure 12. Hence, it is inferred that the greater consistency the pattern has, the lower the number of features and events necessary to define it.

### 7.3. Experiment 2: Pattern Generalization

The objective of this experiment is to revalidate the solution with the highest potential for each participant, which was found by extracting features in EEG A. In this way, it is intended to verify the abstraction or generalization capability of patterns by the trained models.

The best solution for problems A-1, A-2, A-3, and A-4 will be taken and will undergo an evaluation process with an external dataset. That is, the dataset will be different from those used in the training and validation of the corresponding model. Therefore, for this experiment, test sets will be used, which are generated from EEG B.

#### 7.3.1. Results of the Experiment

The results of this experiment are presented in tabular format. For each evaluation, the confusion matrix and the precision (PPV), sensitivity (TPR) and *F*_1_ metrics were calculated.  Table 10 shows the obtained results, where the best performance was achieved with problem B-2 reaching a value of *F*_1_=0.91, while the worst one was with problem B-4 achieving a value of *F*_1_=0.24.

#### 7.3.2. Discussion of the Experiment

Based on the results of Table 10, we can affirm that the model belonging to the best solution of problem A-2 performed better than the rest of the evaluated models. The test set was generated using a window overlap of 87% with the EEG of problem B-2. It is highly probable that the good performance of the model is caused by the fact that it was trained with a large variety of event examples since problem A-2 is the one with the largest number of them. This allowed the model to learn a sufficiently abstract eye-blink pattern, to the point of it being able to successfully detect it in an unknown dataset. As a result, an accuracy PPV=1.0 and a sensitivity TPR=0.84 was obtained, with only nine detection failures out of 57 possible.

On the other hand, the model evaluated with problem B-1 presented a similar performance to that of the model evaluated with problem B-3, just as it happened in the first experiment. Problem B-1 dataset was created with an overlap percentage of 82%, while for problem B-3, a window overlap of 86% was used. The classification results indicate a high incidence of false positives with problem B-1, which significantly affected the accuracy metric and consequently led to a value of *F*_1_=0.82. On the contrary, with problem B-3, a value of *F*_1_=0.88 was achieved with only two false positives and one false negative. From this, we can conclude that the hypothesis regarding the stability of the event pattern is well-founded. The convincing evidence for the aforementioned is the case of problem A-3 ⇒ B-3, where the low number of events in the EEG was compensated by the consistency of the eye-blink action.

The worst performance was presented by the model evaluated with problem B-4, which in the first experiment obtained an accuracy PPV=1.0, a sensitivity TPR=0.87, and an *F*_1_value=0.93. However, in the test set predictions, there were a significant number of false negatives which severely affected the value of the three-evaluation metrics. As a result, an accuracy PPV=0.75, a sensitivity TPR=0.14, and a value *F*_1_=0.24 were obtained. It is evident that there is no agreement between the results of both experiments, thus, we can infer that the model presented overfitting. In addition, another aspect to be considered is the overlap used for the creation of the datasets, which was 11% according to the best solution of problem A-4. In contrast, the best solutions to problems A-1, A-2, and A-3 operate with overlap percentages of 82%, 87%, and 86%, respectively. Therefore, the inference about this fact is that the window overlap should be greater than 80% to avoid loss of information between windows.

## 8. Conclusions

BCI systems aim to satisfy a large part of a person's daily activities by automating tasks, whether for entertainment purposes, work issues, or quality of life, among many others.

In this paper, a methodology to perform EEG feature extraction was proposed, which involves the use of a GA as a search algorithm and an MLP-type ANN as the classification model. To demonstrate the performance of the proposed architecture, eye-blinking action was established as a case study, and a methodology was designed to acquire multiple EEG files containing such an event. Subsequently, two experiments were proposed: the first one aims to perform feature extraction in the acquired EEGs to generate trained models; the second one aims to measure the capability of the trained models in detecting new examples of the event.

The obtained results showed that the proposed method in this paper proved to be successful regarding data dimensionality reduction. In the best case (problem A-3), a decrease of 88% was achieved, whereas in the worst case (problem A-2), it got a decrease of 82.1%.

Likewise, it was observed how the proposed architecture was able to generalize the event within the trained models. The above was demonstrated by evaluating the model with the test dataset. As follows, it was observed that the model is capable to detect the event pattern in most of the presented problems. The obtained results indicate good performance in three out of the four evaluated models, which maintained a metric of *F*_1_ > 0.8.

Additionally, both the dataset and the developed applications are part of a public repository hosted on the GitHub platform. In this repository, the source code of the proposed method implementation is also contained as well as the output files obtained from the performed executions. The aforementioned elements are available in the following URL address: https://github.com/CesarRocha00/evobci.git.

Finally, in the future, it is wished the development of a strategy for automatic event labeling in the acquired EEGs instead of doing it manually. In addition to exploring, extending the proposed architecture for feature extraction in the frequency domain of EEG signals.

## Figures and Tables

**Table 1:** General properties of acquired EEGs.

#	EEG A		EEG B	
	Events	Samples	Events	Samples
1	183	183114	129	60123
2	218	120123	63	63114
3	66	183087	12	60114
4	27	120114	22	63087

**Table 2:** Event distribution for training and validation stages.

ID	Training (70%)		Validation (30%)	
	Events	Samples	Events	Samples
A-1	128	125914	55	57200
A-2	153	88648	65	31475
A-3	46	125185	20	57902
A-4	19	82581	8	37533

**Table 3:** GA execution parameters.

Parameter	Symbol	Value
Population size	*𝒫*	50
Number of generations	*𝒢*	200
Crossover rate	*𝒞*	0.9
Mutation probability	ℳ	Uniform-variable

**Table 4:** Feature selection statistics.

ID	Minimum	Median	Maximum	Mean	*σ*	*ϕ* (%)
A-1	249	634	872	605.00	164.88	69.750
A-2	358	531	924	586.68	133.85	70.666
A-3	240	392	764	437.90	166.40	78.105
A-4	113	243	732	251.48	167.07	87.426

**Table 5:** Nomenclature for the interpretation of the results.

Column	Description
#	Execution number
Overlap (%)	Window overlap percentage
Fp1	Number of selected channel features
Fp2	Number of selected channel features
F3	Number of selected channel features
F4	Number of selected channel features
P1	Number of selected channel features
P2	Number of selected channel features
O1	Number of selected channel features
O2	Number of selected channel features
Total	Sum of selected features
TP	True positives
FP	False positives
TN	True negatives
FN	False negatives
PPV	Precision metric (*positive predictive value*)
TPR	Sensitivity metric (*true positive rate*)
*F*_1_	*F*_1_ metric (*F-score, F-measure*)

**Table 6:** Selected features and metric values of problem A-1.

*Overlap (%)*	*Fp1*	*Fp2*	*F3*	*F4*	*P1*	*P2*	*O1*	*O2*	*Total*

24	128	116	0	113	0	120	0	0	477
87	124	119	0	0	118	0	138	136	635
82	120	129	0	0	0	0	0	0	249

*TP*	*FP*	*TN*	*FN*	*PPV*		*TPR*		*F*_1_	
20	1	225	21	0.952381		0.487805		0.645161	
51	3	1348	0	0.944444		1.000000		0.971429	
46	0	961	3	1.000000		0.938776		0.968421	

**Table 7:** Selected features and metric values of problem A-2.

*Overlap (%)*	*Fp1*	*Fp2*	*F3*	*F4*	*P1*	*P2*	*O1*	*O2*	*Total*

87	136	133	122	124	146	0	131	132	924
87	140	120	0	137	0	134	0	0	531
87	127	108	0	0	123	0	0	0	358

*TP*	*FP*	*TN*	*FN*	*PPV*		*TPR*		*F*_1_	
53	0	483	0	1.0		1.0		1.0	
53	0	483	0	1.0		1.0		1.0	
53	0	483	0	1.0		1.0		1.0	

**Table 8:** Selected features and metric values of problem A-3.

*Overlap (%)*	*Fp1*	*Fp2*	*F3*	*F4*	*P1*	*P2*	*O1*	*O2*	*Total*

30	111	124	129	0	120	119	110	0	713
89	141	121	112	0	0	127	0	0	501
86	119	121	0	0	0	0	0	0	240

*TP*	*FP*	*TN*	*FN*	*PPV*		*TPR*		*F*_1_	
10	0	305	9	1.000000		0.526316		0.689655	
18	0	1885	1	1.000000		0.947368		0.972973	
19	1	1508	0	0.950000		1.000000		0.974359	

**Table 9:** Selected features and metric values of problem A-4.

*Overlap (%)*	*Fp1*	*Fp2*	*F3*	*F4*	*P1*	*P2*	*O1*	*O2*	*Total*

11	0	128	0	0	0	136	0	0	264
87	127	122	120	111	117	119	0	0	716
11	117	121	0	0	0	0	0	0	238

*TP*	*FP*	*TN*	*FN*	*PPV*		*TPR*		*F*_1_	
4	0	157	4	1.000000		0.500		0.666667	
7	0	1107	1	1.000000		0.875		0.933333	
7	0	157	1	1.000000		0.875		0.933333	

**Table 10:** Results of the evaluation with the test set.

ID	TP	FP	TN	FN	PPV	TPR	*F*_1_
B-1	95	40	605	1	0.703704	0.989583	0.822511
B-2	48	0	1482	9	1.000000	0.842105	0.914286
B-3	11	2	1621	1	0.846154	0.916667	0.880000
B-4	3	1	260	18	0.750000	0.142857	0.240000

## Data Availability

The data used to support the findings of this study are included in the article.
